# Construction, complete sequence, and annotation of a BAC contig covering the silkworm chorion locus

**DOI:** 10.1038/sdata.2015.62

**Published:** 2015-11-10

**Authors:** Zhiwei Chen, Junko Nohata, Huizhen Guo, Shenglong Li, Jianqiu Liu, Youbing Guo, Kimiko Yamamoto, Keiko Kadono-Okuda, Chun Liu, Kallare P. Arunkumar, Javaregowda Nagaraju, Yan Zhang, Shiping Liu, Vassiliki Labropoulou, Luc Swevers, Panagiota Tsitoura, Kostas Iatrou, Karumathil P. Gopinathan, Marian R. Goldsmith, Qingyou Xia, Kazuei Mita

**Affiliations:** 1 State Key Laboratory of Silkworm Genome Biology, Chongqing 400716, China; 2 Kirin Brewery Co. Ltd, Toride 302-8502, Japan; 3 National Institute of Agrobiological Sciences, Tsukuba 305-8634, Japan; 4 Centre for DNA Fingerprinting and Diagnostics, Hyderabad 500001, India; 5 Insect Molecular Genetics and Biotechnology, Institute of Biosciences & Applications, National Centre for Scientific Research ‘Demokritos’, Athens 15310, Greece; 6 Indian Institute of Science, Bangalore 560012, India; 7 University of Rhode Island, Kingston, RI 02881, USA

**Keywords:** Molecular biology, DNA sequencing

## Abstract

The silkmoth chorion was studied extensively by F.C. Kafatos’ group for almost 40 years. However, the complete structure of the chorion locus was not obtained in the genome sequence of *Bombyx mori* published in 2008 due to repetitive sequences, resulting in gaps and an incomplete view of the locus. To obtain the complete sequence of the chorion locus, expressed sequence tags (ESTs) derived from follicular epithelium cells were used as probes to screen a bacterial artificial chromosome (BAC) library. Seven BACs were selected to construct a contig which covered the whole chorion locus. By Sanger sequencing, we successfully obtained complete sequences of the chorion locus spanning 871,711 base pairs on chromosome 2, where we annotated 127 chorion genes. The dataset reported here will recruit more researchers to revisit one of the oldest model systems which has been used to study developmentally regulated gene expression. It also provides insights into egg development and fertilization mechanisms and is relevant to applications related to improvements in breeding procedures and transgenesis.

## Background & Summary

Silkmoth chorion proteins, the main components of the eggshell, are sequentially synthesized and secreted by follicular epithelium cells with a high degree of developmental programming^1^. The structural genes for chorion proteins comprise a multigene family whose members are grouped under α and β branches based on their evolutionarily conserved central domains^2^. Chorion proteins are further classified into six subgroups, early A, early B, middle A, middle B, late high-cysteine A (HcA) and late high-cysteine B (HcB), according to their timing of developmental expression and amino acid composition^3^. Based on genetic linkage mapping, the chorion genes are located between the larval marker *p* at the proximal end of chromosome 2 and the cocoon color marker *Y*^4–6^. The recent silkworm genome assembly^7^ indicates that the chorion locus is localized at [1,780,900–3,840,078] on chromosome 2, although it is largely interrupted by gaps due to highly repetitive sequences.

**Figure 1:**
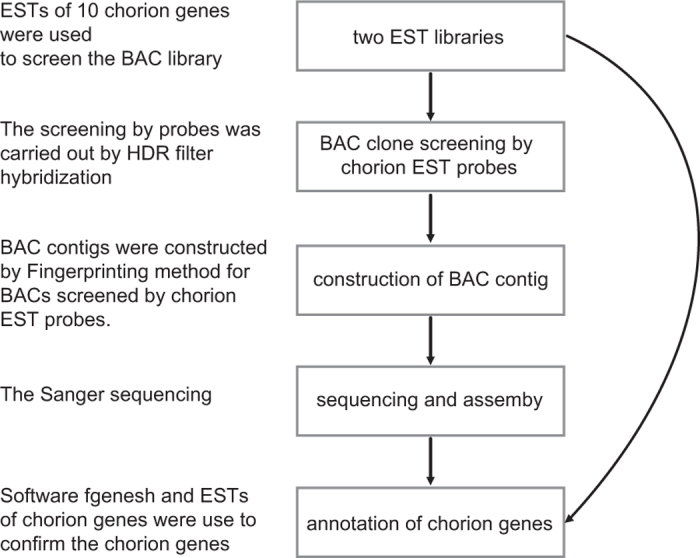
Schematic overview of the study.

**Figure 2:**
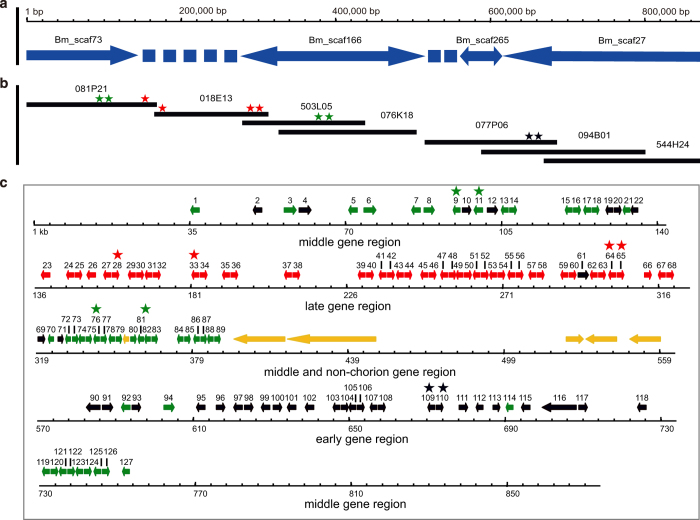
Distribution of genome assembly, BAC contig and annotated chorion genes in the chorion locus. Probes are marked by stars: early chorion genes (black stars); middle chorion genes (green stars); late chorion genes (red stars). The probes used here are presented in Table 1. (**a**) Diagram of the chorion locus in the *B*. *mori* genome assembly. Arrows and dotted lines represent scaffolds and gap regions, respectively, edited from KAIKObase, respectively. (**b**) BAC contig that covers the chorion locus. Each black line represents a complete BAC region. Six BACs were sequenced except for 544H24, because its sequence was known. (**c**) Early, middle, late and non-chorion genes are highlighted in black, green, red and yellow, respectively.

A high quality BAC library was constructed from genomic DNA of silkworm fifth instar day 3 posterior silk glands partially digested with *Eco*RI^8^, designated RPCI-96 (RP96), and is available from BACPAC Resources of the Children’s Hospital Oakland Research Institute (BACPAC Resources Center [bacpac.chori.org/]). Here we undertook the following strategy to obtain complete sequences of the chorion locus (Fig. 1): ESTs of chorion genes were used as probes to screen the BAC library, and selected clones were used to construct a BAC contig which covered the complete chorion locus (Fig. 2b). By Sanger sequencing of the BAC contig, we successfully obtained the complete sequence of the chorion locus spanning 871,711 base pairs on chromosome 2, where we annotated 127 chorion genes (Fig. 2c).

We report and describe in detail the methods, data and quality measurements for the construction and sequencing of the silkmoth chorion BAC contig in this paper. Additional information for a comprehensive understanding of the structure, transcription, and proteomics of genes in the chorion locus is described in a related research paper^9^. In the present paper, we describe in detail our experimental approach for obtaining the complete BAC contig covering the silkworm chorion locus, together with its sequence data and annotation, which are presented briefly in the ‘Materials and Methods’ section of our related paper^9^. Our strategy can serve as a model to facilitate the sequencing of selected loci in genomes in other species containing highly repetitive sequences.

## Methods

### EST analysis of follicular cell and ovary cDNA libraries

To identify chorion gene transcripts, we analyzed ESTs of two newly constructed cDNA libraries, fcP8 derived from day 8 pupal follicular cells and bmov from day 4 pupal ovaries. All ESTs derived from the bmov and fcP8 cDNA libraries are accessible at the DNA Database of Japan (acc # FY000001-FY021573 for bmov and BY918786-BY920388 and BY927072-BY928825 for fcP8). We identified ESTs of chorion genes by BLASTx search in public protein databases including nr of NCBI.

### BAC screening

The silkworm BAC library (RPCI-96) used in this paper was obtained from BACPAC Resources Center, Children’s Hospital, Oakland Research Institute and previously described^8,10^. BAC clones derived from the chorion locus were screened by hybridization of BAC high density replica (HDR) filters arrayed in duplicate with RPCI-96 BAC clones (BACPAC Resources Center [bacpac.chori.org/]) using the ESTs of 10 chorion genes selected as representatives of the three chorion families which provided strong signals in hybridization with multiple BACs, among which some were cross-hybridized with different chorion families. A list of ESTs used for BAC screening is presented in Table 1. Labeling, hybridization and detection were performed using the ECL Direct Nucleic Acid Labeling and Detection System (GE Heathcare UK Ltd., Little Chalfont, Buckinghamshire, UK), in accordance with the manufacturer’s instructions^8^.

### Construction of a BAC contig covering the chorion locus

Two hundred and two BAC clones from early, middle and late chorion gene regions were screened with EST probes of representative chorion genes from the fcP8 cDNA library by hybridization of an HDR filter of the RPCI-96 silkworm BAC library. Among positive BAC clones, we chose highly positive BAC clones 077P06 and 094B01 for early chorion genes, 081P21 and 076K18 for middle chorion genes, and 018E13 for late chorion genes. We also selected clone 503L05, which had a strong positive signal and was known to cover a non-chorion domain of the locus based on its BAC end sequence, BES_503_L05 (acc # DE379518), in (http://sgp.dna.affrc.go.jp/KAIKObase/), and BAC 544H24, because we already knew that its full sequence was aligned with the 3′ part of the chorion locus and the neighboring region^7^. We performed contig construction for these BAC clones with the fingerprinting method described previously^10^. This resulted in two contigs; one was composed of four BACs covering the 5′ half of the chorion locus, while the other was composed of three BACs aligning with the 3′ half of the chorion locus (Fig. 2a). One of the 076K18 BAC-end sequences, BES_076_K18 (acc # DE307437), aligned to Bm_scaf166 at [chr2: 2,636,193-2,636,430], and the 5′ end of the other BAC contig, 077P06 BAC end-sequence BET_077_P06 (acc # DE354956), was located on the same scaffold, Bm_scaf166, at [chr2: 2,647,297-2,647,961]. Thus, the two BAC contigs, which were connected on Bm_scaf166, covered the whole chorion locus (Fig. 2a).

### Genomic sequencing

Six BAC clones from 384 well plates^11^ were streaked separately on chloramphenicol-containing LB plates. Three single clones from each plate were checked to confirm the correct BAC clone by using primers designed from the end sequences of each BAC (Table 2). Then BAC clones were cultured for isolation of BAC DNA in LB medium. BAC DNA was extracted using a Large-Construct Kit (QIAGEN) in accordance with the manufacturer’s instructions. Two kilobase and five kilobase shotgun libraries for each BAC were constructed using a pUC118 vector^12^. For each library, approximately 590 clones were picked for bidirectional sequencing performed with an ABI3730 DNA Analyzer (Applied Biosystems).

### Sequence assembly and annotation of chorion genes

The low-quality bases (QV<20) were removed by Phred^13^. After trimming vector sequences using cross_match, all paired-end reads were assembled with the programs Phrap 1.08081222^14^ and Consed 16.0^15^. The position of mis-assembled clone sequences could be adjusted according to the size of the clones (insertion segment) by both assembly programs. The small gap in assembly sequences was filled by primer walking. The software program fgenesh^16^ was used to predict the chorion genes.

## Data Records

### Data record 1

The complete sequence of the chorion locus appears under *DDBJ* AB999997 (Data Citation 1).

## Technical Validation

### Probe selection and construction of BAC contig

Previous reports revealed that the chorion locus is composed of three types of clusters containing early, middle and late chorion genes^3^. Thus, we selected representatives for the three types of chorion gene ESTs to screen the BAC library (Table 1). Among ten probes, eight of them were identified and oriented in the published genome of *B. mori*^7^, and both end sequences of BACs were used to confirm the orientation of BACs. BAC end sequence-based primers were used to confirm the orientation and position of BACs in the chorion locus by PCR (Supplementary Fig. 1; see Table 2 for primer sequences). The PCR experiment showed that the target BACs were sequentially connected with an overlap to cover the whole chorion locus, except for a small gap region. Then, we were able to obtain sequences for the gap region between BACs 076K18 and 077P06 from Bm_scaf166 in the silkworm genome sequence. These strategies enabled us to establish a complete BAC contig covering the chorion locus.

### Sequencing and assembly

In a first attempt to obtain the complete sequence of the chorion locus, we used Ion PGM^TM^, a representative of a second generation sequencing platform characterized by low cost, high throughput and read lengths of up to 289 bp. Unfortunately, the presence of highly repetitive DNA sequences resulted in a failure to obtain an assembly of individual BACs despite a coverage of 150-fold. For further assistance in sequence assembly, we constructed 2 and 5 kb shotgun libraries for each BAC and sequenced them using the Sanger method. This enabled the generation of reads up to 500 bp, which were able to cover major exons of chorion genes, on the order of 500–800 bp. About 2,400 reads were generated for each BAC, which covered the chorion locus 10-fold. The positions of the BACs in the complete chorion locus are shown in Table 3.

### Annotation of chorion genes

Two EST libraries from day 4 pupal ovary and day 8 pupal follicular cells were constructed which contained ESTs of all known chorion genes. ESTs were aligned to the chorion locus, which further confirmed the existence of the predicted chorion genes.

## Usage Notes

The complete sequences of chorion locus data described here can be downloaded from *DDBJ* AB999997. This data descriptor provides an opportunity to present a strategy for obtaining precise sequence information for an extended region (>0.8 Mb) of a highly repetitive genome. The complete sequence of the chorion locus and detailed gene annotation data are provided for users to study developmental regulation of gene expression using the silkmoth chorion gene model.

## Additional Information

**How to cite this article:** Chen, Z. *et al.* Construction, complete sequence, and annotation of a BAC contig covering the silkworm chorion locus. Sci. Data 2:150062 doi: 10.1038/sdata.2015.62 (2015).

## Supplementary Material

Supplementary Information

## Figures and Tables

**Table 1:** ESTs used as probes for screening BAC clones.

**Gene ID**	**cDNA clone**	**Accession #**	**Type of chorion**	**BAC clone #**
BmCho-9	fcP812C07	BY919605	middle class A	081P21
BmCho-11	fcP809B08	BY919370	middle class B	081P21
BmCho-28	fcP802B12	BY927183	late HcA	081P21
BmCho-33	fcP815F06	BY919864	late HcB	018E13
BmCho-64	fcP816D07	BY919923	late HcA	018E13, 503L05
BmCho-65	fcP806G06	BY919206	late HcB	018E13, 503L05
BmCho-76	fcP814C12	BY919762	middle class A	503L05, 076K18
BmCho-82	fcP818B03	BY920045	middle class B	503L05, 076K18
BmCho-109	fcP807G07	BY927624	early B	077P06, 049B01
BmCho-110	fcP809F08	BY919409	early A	077P06, 049B01

**Table 2:** The list of primers for detecting the BAC clones

**BAC End**	**Forward Primer**	**Reverse Primer**
BET_081P21	AGCATTCTTCCCCCACTGA	GATTTAGATAGGCGGACGAA
BET_018E13	CATCCACTGTAACCTCCATA	TACAGAGCAAGTGGATTTTC
BES_018E13	AGCCACGTTTCTTCCAATCA	TGAGGATGTGGTGTCAAACG
BET_503L05	TTTTCCGAATTTAAGCGAT	AGTGGAGTCAAAAAGTAGATGT
BES_503L05	GCACAGTAATTCGCCAGTAG	GCTGCCATTGACCTGATAGA
BES_076K18	TAGTTATTCTACGCAGTTCAGG	GGAGGTCTATGTCCAGCGG
BET_077P06	ATTTTTATCCGACACCCTTA	TTCCCGCCAAAAAGTCATAC
BES_077P06	GCGCATTTACGATGTAGATG	CAATGTATGTTCCGCTGTGT
BET_094B01	CTTAACGCAATTCGTCGGTA	GGAAAGGTCACCTACGAATG
BES_094B01	AAGCAACTCTTTTACGGGTC	ATTAGATAAATGAAGGCCGG
Note: BET is a BAC-end sequence using a T7 primer; BES uses a SP6 primer. Both are vector primers which align adjacent to an insert. BET primer >Insert<BES primer.		

**Table 3:** BACs and their position in the complete chorion locus

**BAC**	**Position in chorion locus (nt)**
081P21	1–169,292
018E13	165,668–313,154
503L05	279,542–438,320
076K18	326,219–505,371
077P06	515,752–687,226
049B01	592,429–801,223
544H24	669,876–871,711
Note: BET is a BAC-end sequence using a T7 primer; BES uses a SP6 primer. Both are vector primers which align adjacent to an insert. BET primer>Insert<BES primer.	

## References

[d1] DDBJ Mita K. Chen Z. Xia Q. Kadono-Okuda K. 2015 AB999997

