# Supplementary material for: Construction, complete sequence, and annotation of a BAC contig covering the silkworm chorion locus
Source: Sci Data. 2015 Nov 10;2:150062. doi: 10.1038/sdata.2015.62 (PMC4640134; doi:10.1038/sdata.2015.62)
Supplement: Supplementary Information [file sdata201562-s1.doc]

**Construction, Complete Sequence, and Annotation of a BAC Contig Covering the Silkworm Chorion Locus**

Zhiwei Chen^1^, Junko Nohata^2^, Huizhen Guo^1^, Shenglong Li^1^, Jianqiu Liu^1^, Youbing Guo^1^, Kimiko Yamamoto^3^, Keiko Kadono-Okuda^3^, Chun Liu^1^, Kallare P. Arunkumar^4^, Javaregowda Nagaraju^4✝^, Yan Zhang^1^, Shiping Liu^1^, Vassiliki Labropoulou^5^, Luc Swevers^5^, Panagiota Tsitoura^5^, Kostas Iatrou^5*^, Karumathil P. Gopinathan^6^, Marian R. Goldsmith^7*^, Qingyou Xia^1^ & Kazuei Mita^1*^

^1^State Key Laboratory of Silkworm Genome Biology, Chongqing 400716, China. ^2^Kirin Brewery Co. Ltd, Toride, Japan. ^3^National Institute of Agrobiological Sciences, Tsukuba 305-8634, Japan. ^4^Centre for DNA Fingerprinting and Diagnostics, Hyderabad 500001, India. ^5^Insect Molecular Genetics and Biotechnology, Institute of Biosciences & Applications, National Centre for Scientific Research “Demokritos”, Athens 15310, Greece. ^6^Indian Institute of Science, Bangalore, India. ^7^University of Rhode Island, Kingston 02881, USA.

^✝^ deceased

**Corresponding authors**:

^1^State Key Laboratory of Silkworm Genome Biology, Chongqing 400716, China. E-mail: mitakazuei@gmail.com. Tel: +86-18983794244. Fax: +86-023-68250892; ^7^Department of Biological Sciences, University of Rhode Island, Kingston, Rhode Island 02881. E-mail: mki101@uri.edu. Tel: 1-401-874-2637; and ^5^Insect Molecular Genetics and Biotechnology, Institute of Biosciences & Applications, National Centre for Scientific Research “Demokritos”, Athens 15310, Greece. E-mail: iatrou@bio.demokritos.gr. Tel: +30-210-650-3562; Fax: +30-210-651-1767


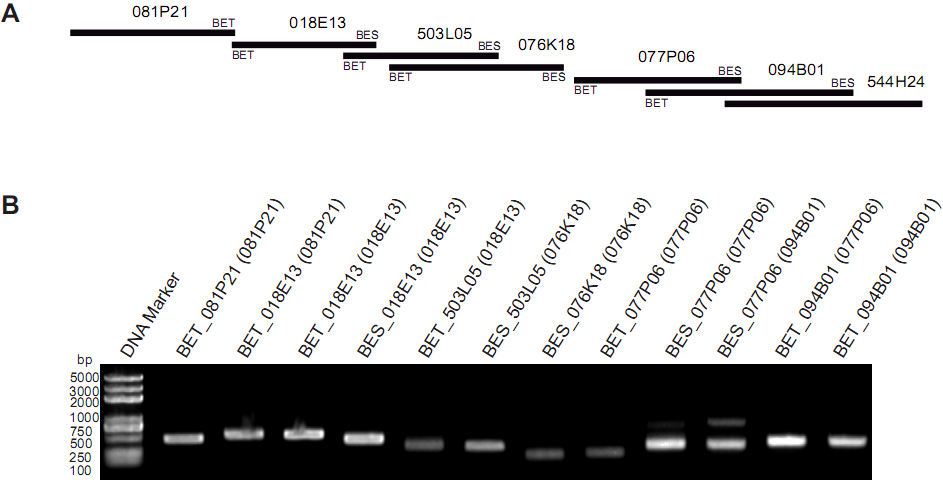
**Supplementary information, figure S1**

**Supplementary Figure 1**. The validation of orientation and position of BACs in BAC contigs of chorion locus by PCR. **A**. BAC contig that covers the chorion locus, and the end of BAC was marked by BES and BET. **B**. The detection of BAC clones. The PCR primer was marked by the title out of brackets for each lane. BAC clone in brackets was used as template in PCR reaction.
